# Phosphate (Pi) Starvation Up-Regulated *GmCSN5A*/*B* Participates in Anthocyanin Synthesis in Soybean (*Glycine max*) Dependent on Pi Availability

**DOI:** 10.3390/ijms222212348

**Published:** 2021-11-16

**Authors:** Xiaohui Mo, Mengke Zhang, Zeyu Zhang, Xing Lu, Cuiyue Liang, Jiang Tian

**Affiliations:** Root Biology Center, State Key Laboratory for Conservation and Utilization of Subtropical Agro-Bioresources, College of Natural Resources and Environment, South China Agricultural University, Guangzhou 510642, China; xhmo@scau.edu.cn (X.M.); mengkezhang611@foxmail.com (M.Z.); jysx@stu.scau.edu.cn (Z.Z.); xinglu@scau.edu.cn (X.L.)

**Keywords:** phosphorus deficiency, soybean, *GmCSN5A/B*, anthocyanin synthesis

## Abstract

Phosphorus (P) is an essential macronutrient for plant growth and development. Among adaptive strategies of plants to P deficiency, increased anthocyanin accumulation is widely observed in plants, which is tightly regulated by a set of genes at transcription levels. However, it remains unclear whether other key regulators might control anthocyanin synthesis through protein modification under P-deficient conditions. In the study, phosphate (Pi) starvation led to anthocyanin accumulations in soybean (*Glycine max*) leaves, accompanied with increased transcripts of a group of genes involved in anthocyanin synthesis. Meanwhile, transcripts of *GmCSN5A*/*B*, two members of the *COP9 signalosome subunit 5* (*CSN5*) family, were up-regulated in both young and old soybean leaves by Pi starvation. Furthermore, overexpressing *GmCSN5A* and *GmCSN5B* in *Arabidopsis thaliana* significantly resulted in anthocyanin accumulations in shoots, accompanied with increased transcripts of gene functions in anthocyanin synthesis including *AtPAL*, *AtCHS*, *AtF3H*, *AtF3*′*H*, *AtDFR*, *AtANS*, and *AtUF3GT* only under P-deficient conditions. Taken together, these results strongly suggest that P deficiency leads to increased anthocyanin synthesis through enhancing expression levels of genes involved in anthocyanin synthesis, which could be regulated by GmCSN5A and GmCSN5B.

## 1. Introduction

Phosphorus (P) is one of essential mineral nutrients required for various biomacromolecules (e.g., nucleic acids and phospholipids), and a series of the biological processes, such as cell energy metabolism, signal transduction and protein modification in plants [1,2,3]. Inorganic phosphate (Pi) is the main form of phosphorus absorbed by plants, and is easily fixed in soils, mainly due to cation chelation and biological fixation by microorganisms [1,2]. Therefore, the amount of available Pi is approximately between 1 μM and 10 μM, which is suboptimal for crop growth [4,5]. Plants have deployed a set of morphological and physiological strategies in adaptation to P deficiency, including retardation of growth rates, enhancement of lateral root and root hair growth, accumulations of anthocyanin [6,7]. Meanwhile, plants have evolved the metabolic flexibility to improve P utilization efficiency, such as scavenging Pi from P-containing metabolites, replacing membrane P-lipids with sulfonyl-lipids [3,8]. Among them, anthocyanin accumulation in shoots or leaves of *Arabidopsis thaliana*, tomato (*Solanum lycopersicum*), tobacco (*Nicotiana tabacum*), *Medicago truncatula*, rice (*Oryza sativa*) and maize (*Zea mays*) was widely observed and suggested to protect chloroplasts from photoinhibitory damage in plant responses to Pi starvation [7,9,10,11,12,13].

Anthocyanins are a kind of flavonoids, and exist in most plant tissues, including flower petals, leaves, stems, roots, and fruits [14]. Anthocyanins not only act as the pigments in tissues or organs (e.g., flowers, fruits, and leaves), but also protect plants from pathogen and insect invasion [15,16,17]. Meanwhile, the antioxidant activity of anthocyanins helps plants adapt to various abiotic stresses, such as nutrient deficiencies (e.g., nitrogen and phosphorus), and low temperature stress [18,19,20]. In Arabidopsis, biosynthesis of anthocyanins includes consecutive processes which are catalyzed by multiple enzymes including phenylalanine ammonia-lyase (PAL), chalcone synthase (CHS), chalcone isomerase (CHI), flavanone-3-hydroxylase (F3H), flavonoid 3′-hydroxylase (F3′H), dihydroflavonol 4-reductase (DFR), anthocyanin synthase (ANS), and flavonoid 3-*O*-glucosyl transferase (UF3GT) [21,22]. Therefore, the synthesis of anthocyanins is tightly related to enzymatic activity of the enzymes, and transcription levels of their corresponding genes in plants [7,23]. For example, anthocyanin synthase (ANS) is the key enzyme in anthocyanin synthetic pathways, and *ans-1*/*2* mutants accumulate less anthocyanin under high light stress in Arabidopsis [24].

Except for the functional identification of gene functions in anthocyanin biosynthesis processes, their upstream regulatory factors have been well characterized in plants [1,25,26]. Among them, the MYB-bHLH-WD40 (MBW) complexes are considered to play a critical role in controlling transcription levels of genes encoding anthocyanin biosynthesis [27,28,29,30,31]. In these complexes, a R2R3 MYB transcription factor PRODUCTION OF ANTHOCYANIN PIGMENTS1 (PAP1), also known as MYB75, functions as central regulator in anthocyanin biosynthesis in plants [30,31]. Therefore, it is reasonable to find that increased anthocyanin accumulations by Pi starvation were regulated by *PAP1* in plants, such as Arabidopsis [29,31]. Recently, SPX4, the key regulator of Pi signaling network, has been found to cooperate with PAP1, and thus transform the signal of P deficiency to anthocyanin biosynthesis in plants [26].

Although the molecular mechanisms underlying anthocyanin synthesis are well demonstrated at transcription levels in model plants (i.e., Arabidopsis and rice), it remains largely unknown that protein modification might be involved in mediating the anthocyanin synthesis in plants. It is well known that a ubiquitin-proteasome-mediated protein degradation is involved in photomorphogenesis, floral organ formation and abiotic stress responses [32,33,34]. Among them, constitutive photomorphogenesis 9 (COP9) signalosome (CSN) was found to participate in protein degradation, mainly through interacting with Skp1-cullin 1-F-box (SCF) complexes, the largest family of ubiquitin E3 ligases [35]. Furthermore, loss-function of *CSN5a*, one of the eight CSN subunits, was found to enhance anthocyanin accumulations in Arabidopsis, strongly suggesting that AtCSN5A participates in anthocyanin synthesis [36,37,38]. However, the molecular mechanisms underlying CSN5A mediating anthocyanin metabolism in other plants, remain largely unknown especially in crops.

Soybean (*Glycine max*) is an important legume crop, and it provides a protein-rich food source in the world. However, phosphate starvation restrains soybean growth and yield in soils [39]. To deal with low Pi stress, the soybean has evolved a set of adaptive strategies to enhance Pi mobilization and acquisition in soil, such as the remodeling of root architecture, increased exudation of organic acids and root-associated purple acid phosphatase activities, formation of symbiosis with beneficial microbes (i.e., rhizobia and mycorrhiza fungi) [40,41,42,43]. However, few studies tried to investigate responses of soybean leaves to P deficiency in order to fully understand adaptative strategies of soybean to P deficiency. Therefore, in the study, we examined anthocyanin accumulations in both old and young soybean leaves, as well as transcripts of anthocyanin synthesis-related genes under Pi-sufficient and Pi-deficient conditions. Meanwhile, two Pi starvation up-regulated *GmCSN5A*/*B* members were functionally characterized, and were suggested to regulate anthocyanin synthesis tightly dependent on Pi availability through mediating transcription levels of several genes related to anthocyanin synthesis in plants.

## 2. Results

### 2.1. Effects of P Deficiency on Soybean Biomass and Anthocyanin Content

**Figure 1:**
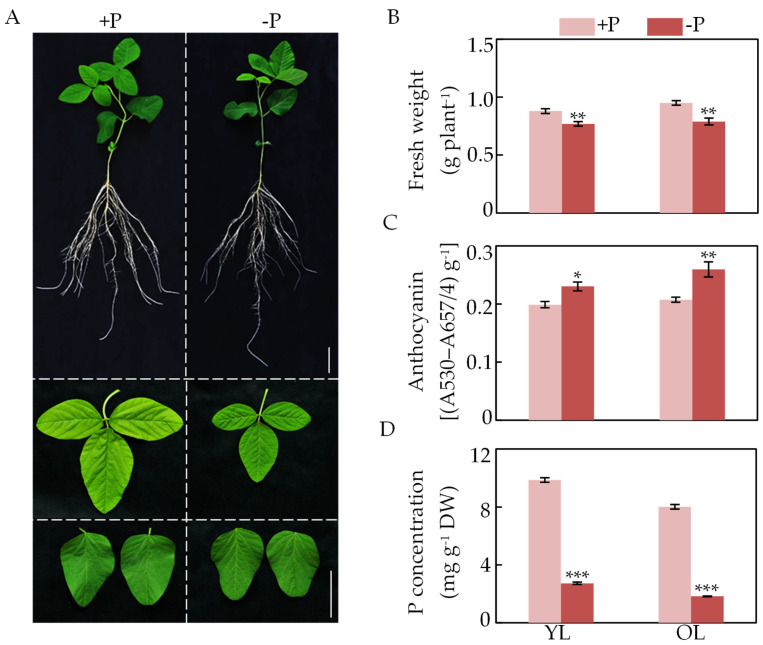
Effects of P deficiency on soybean fresh weight, anthocyanin content and P concentration. (**A**) Phenotypes of soybean at two P levels. Bars = 5 cm. (**B**) Fresh weight of young leaves (YL) and old leaves (OL). (**C**) Anthocyanin content of YL and OL. (**D**) P concentration of YL and OL. Data are means of four replicates ± SE. Asterisks indicate significant difference between the two P levels in the Student’s *t*-test (* *p* < 0.05; ** *p* < 0.01; *** *p* < 0.001).

To investigate the effects of P deficiency on soybean growth, the fresh weight of leaves at two P treatments for 12 d was measured. The results showed that the fresh weight of young and old leaves under sufficient P conditions was 12.5% and 16.8% higher than that of P-deficient plants, respectively (Figure 1A,B). Anthocyanin content in both young and old leaves was determined at two P levels. As shown in Figure 1C, the anthocyanin content in young and old leaves of soybean seedlings grown under Pi-deficient conditions was about 1.2 and 1.3 times higher than those of plants grown on Pi-sufficient conditions, respectively. However, P concentration in young and old leaves was 72.3% and 77.2% lower at low P levels than that at high P levels, respectively (Figure 1D). These results suggest that Pi starvation led to increased accumulations of anthocyanin in soybean leaves.

### 2.2. Effects of P Deficiency on Transcripts of Anthocyanin Synthesis Genes

**Figure 2:**
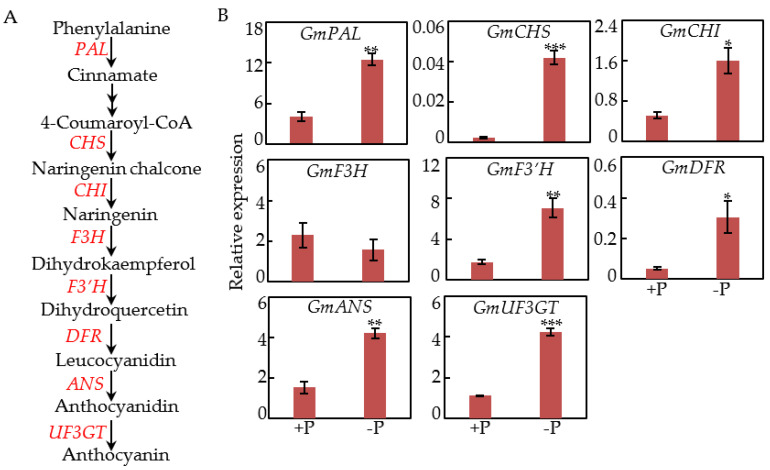
Transcripts of eight genes in anthocyanin biosynthetic pathway in soybean leaves at two P levels. (**A**) The genes in anthocyanin biosynthetic pathway. (**B**) Relative expression of genes in anthocyanin biosynthetic pathway under Pi-sufficient (+P) and Pi-deficient (−P) conditions. Data are means of four independent replicates ± SE. Asterisks indicate significant difference between the two P levels in the Student’s *t*-test (* *p* < 0.05; ** *p* < 0.01; *** *p* < 0.001).

In order to analyze the effects of Pi starvation on anthocyanin synthesis in soybean, the expression patterns of eight genes related to anthocyanin synthesis pathway were investigated in old leaves under two P treatments, including *phenylalanine ammonia*-*lyase* (*GmPAL*), *chalcone synthase* (*GmCHS*), *chalcone isomerase* (*GmCHI*), *flavanone*-*3*-*hydroxylase* (*GmF3H*), *flavonoid 3*′-*hydroxylase* (*GmF3*′*H*), *dihydroflavonol* *4*-*reductase* (*GmDFR*), *anthocyanin synthase* (*GmANS*) and *flavonoid* *3*-*O*-*glucosyl transferase* (*GmUF3GT*) (Figure 2A). Results showed that transcripts of all genes were enhanced by more than 1-fold by Pi starvation, except *F3H,* which exhibited no response to P treatments (Figure 2B). Notably, the expression levels of *GmDFR* under low P conditions were 5.8-fold higher than those under high P conditions (Figure 2B).

### 2.3. Identification of GmCSN5A/B in Soybean Genome

**Figure 3:**
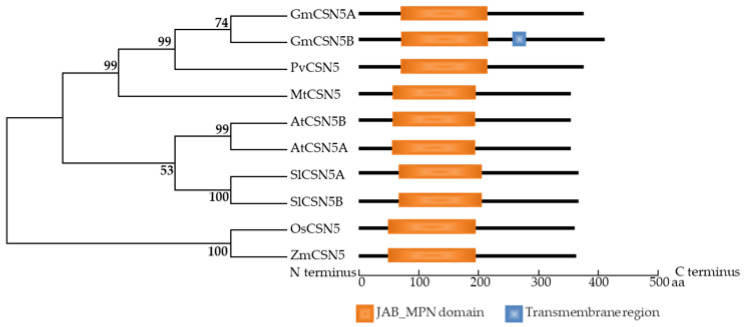
Phylogenetic analysis of CSN5s in plant. The phylogenetic tree was generated ground on an amino acid alignment of CSN5 proteins from well-known species using the Mega 5.05 program. The gene loci on the Phytozome website are as follows: AtCSN5A (At1G22920), AtCSN5B (At1G71230), GmCSN5A (Glyma.04G075000), GmCSN5B (Glyma.06G076000), PvCSN5 (Phvul.009G100700), MtCSN5 (Medtr3g108790), SlCSN5A (Solyc06g073150), SlCSN5B (Solyc11g017300), OsCSN5 (LOC_Os04g56070), ZmCSN5 (GRMZM2G086801). At, *Arabidopsis thaliana*; Gm, *Glycine max*; Os, *Oryza sativa*; Pv, *Phaseolus vulgaris*; Zm, *Zea mays*; Mt, *Medicago truncatula*; Sl, *Solanum lycopersicum*.

Two putative GmCSN5 members in the soybean genome were identified by BLAST searching at http://www.phytozome.net, accessed on 10 February 2019, which were named as *GmCSN5A* (Glyma.04G075000) and GmCSN5B (Glyma.06G076000) based on its localization in soybean chromosomes. All the tested CSN5 proteins contained the conserved JAB_MPN domain (Figure 3). Phylogenetic analysis showed that CSN5 homologs in dicot plants significantly differed from monocot plants (Figure 3). Meanwhile, among dicot plants, CSN5 homologs in leguminous plants distinguished from other dicot plants, as reflected by CSN5 homologs from soybean, bean and Medicago, belonged to a subgroup. Interestingly, CSN5B was found to contain a transmembrane region, which was not identified in other CSN5 homologs (Figure 3).

### 2.4. Expression Patterns and Subcellular Localization of GmCSN5A/B

**Figure 4:**
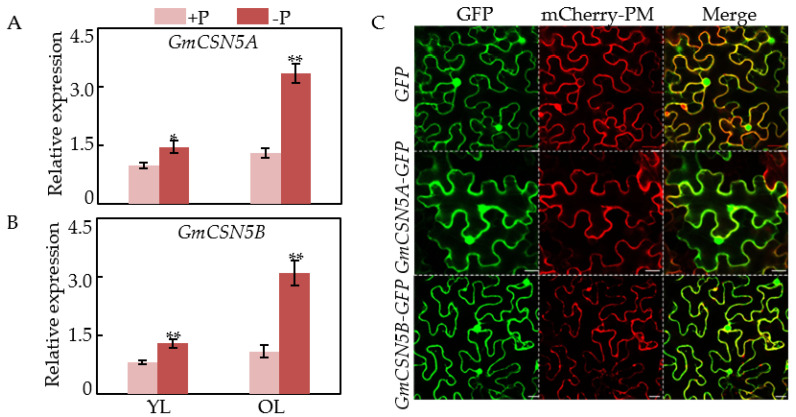
Expression patterns and subcellular localization of *GmCSN5A/B*. (**A**) Relative expression of *GmCSN5A*. (**B**) Relative expression of *GmCSN5B*. Data are mean of four replicates ± SE. Asterisks indicate significant difference between the two P levels in the Student’s *t*-test (* *p* < 0.05; ** *p* < 0.01). (**C**) Subcellular localization of *GmCSN5A* and *GmCSN5B*. mCherry-PM represents plasma membrane localization indicated by the red fluorescence derived from AtPIP2A-mCherry. Bars = 20 µm.

Expression patterns of *GmCSN5A* and *GmCSN5B* in leaves were investigated at two P levels using qRT-PCR. Results showed that transcripts of *GmCSN5A* in young and old leaves were significantly enhanced by low P stress, as reflected by 1.5-fold and 2.6-fold increases, respectively (Figure 4A). Similarly, transcripts of *GmCSN5B* in leaves were also up-regulated by Pi starvation, especially in old leaves with values 2.8-fold higher than those in sufficient P treatment (Figure 4B). These results suggest that *GmCSN5A* and *GmCSN5B* exhibited enhanced expression patterns in soybean leaves in response to Pi starvation.

To analyze the subcellular localization of GmCSN5A/B, the *GFP* (empty vector control), *GmCSN5A-GFP* and *GmCSN5B-GFP* plasmids were separately transiently expressed in tobacco leaf epidermal cells. Results showed that GmCSN5A-GFP fluorescence signals were detected in the cytoplasm and nucleus, and GmCSN5B-GFP fluorescence signals were detected in the whole epidermal cells, exhibiting similar patterns to the GFP fluorescence signals observed in cells transformed with the empty vector (Figure 4C). Therefore, it is suggested that GmCSN5A is mainly localized in the cytoplasm and nucleus, while GmCSN5B is localized in the plasma membrane, nucleus, and cytoplasm.

### 2.5. Overexpression of GmCSN5A/B Increases Anthocyanin Accumulations and Affects Photomorphogenesis in Arabidopsis

**Figure 5:**
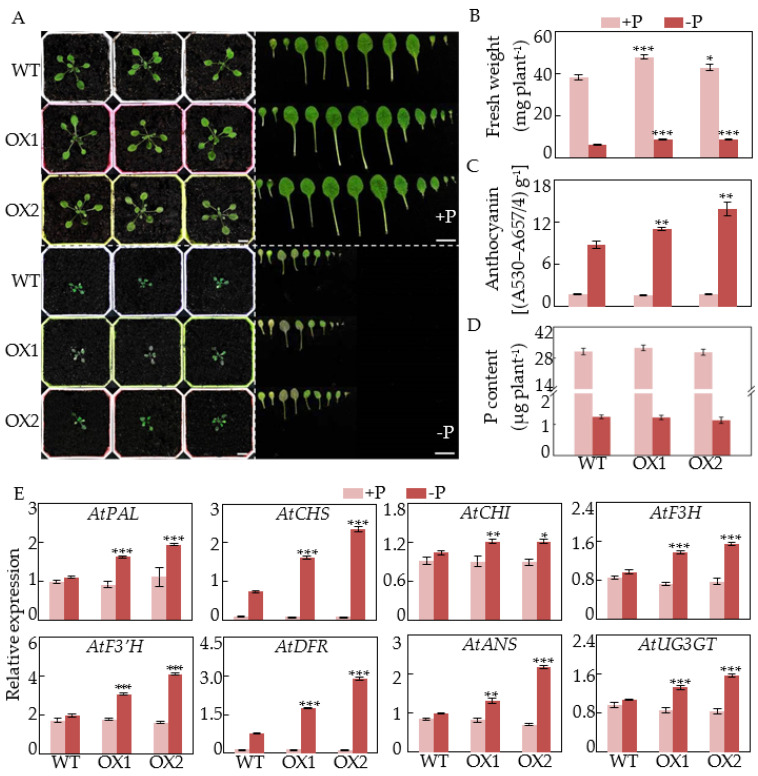
Effects of *GmCSN5A* overexpression on anthocyanin accumulation in Arabidopsis. (**A**) The phenotypes. Bars = 1 cm. (**B**) Fresh weight. (**C**) Anthocyanin content. (**D**) P content. (**E**) Relative expression of eight genes in anthocyanin biosynthetic pathway. Seedlings were grown in peat soils for 8 d. Data are means of four independent replicates ± SE. Asterisks indicate significant difference between *GmCSN5A* overexpressing Arabidopsis and WT in the Student’s *t*-test (* *p* < 0.05; ** *p* < 0.01; *** *p* < 0.001).

**Figure 6:**
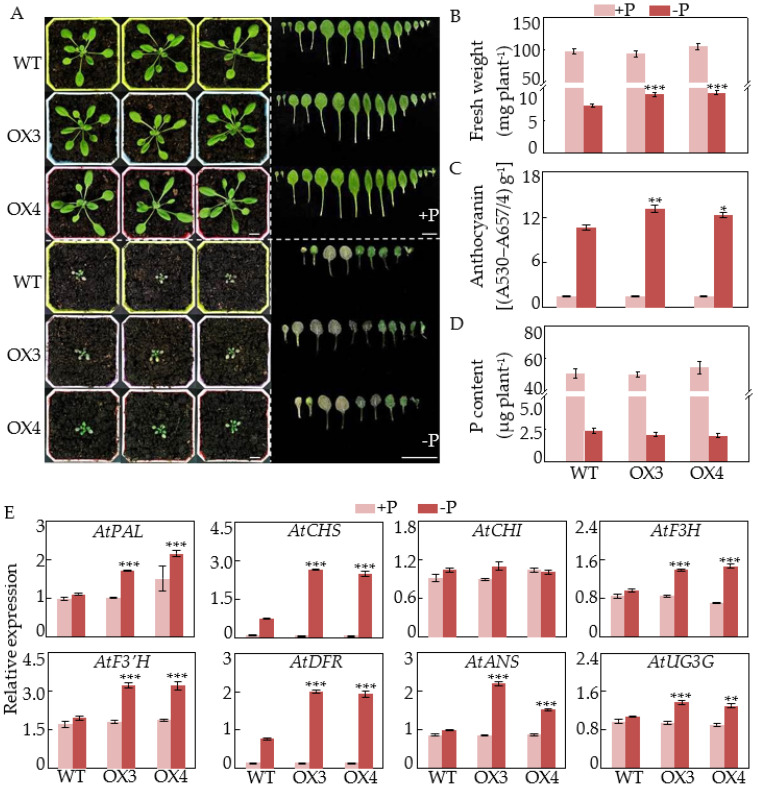
Effects of *GmCSN5B* overexpression on anthocyanin accumulation in Arabidopsis. (**A**) The phenotypes. Bars = 1 cm. (**B**) Fresh weight. (**C**) Anthocyanin content. (**D**) P content. (**E**) Relative expression of eight genes in anthocyanin biosynthetic pathway. Seedlings were grown in peat soils for 13 d. Data are means of four independent replicates ± SE. Asterisks indicate significant difference between *GmCSN5B* overexpressing Arabidopsis and WT in the Student’s *t*-test (* *p* < 0.05; ** *p* < 0.01; *** *p* < 0.001).

In this study, *GmCSN5A* and *GmCSN5B* were subsequently overexpressed in Arabidopsis to investigate their biological functions. Increased transcripts of *GmCSN5A* and *GmCSN5B* in transgenic Arabidopsis were validated via qRT-PCR analysis (Figure S1). Subsequently, wild type (WT) and transgenic Arabidopsis with overexpressing *GmCSN5A*/*B* were used to investigate the role of *GmCSN5A*/*B* in anthocyanin accumulations at two P levels (Figure 5 and Figure 6).

Wild type (WT) and *GmCSN5A* transgenic Arabidopsis were subjected to two P treatments for 8 d. Results showed that *GmCSN5A* overexpression resulted in enhanced Arabidopsis growth at two P levels. Fresh weight in two *GmCSN5A* overexpression lines (OX1 and OX2) was 12.3–25.3% and 39.6–40.1% higher than that in WT under P-sufficient and P-deficient conditions, respectively (Figure 5B). Furthermore, under low P conditions, anthocyanin content in *GmCSN5A* overexpression lines was increased by approximately more than 1.4-fold compared to those in WT (Figure 5C). For P content, no significant difference was observed between *GmCSN5A* overexpression lines and WT at two P levels (Figure 5D). To further investigate the roles of *GmCSN5A* in anthocyanin synthesis in responses to Pi starvation, transcripts of eight genes involved in the anthocyanin synthesis pathway were analyzed in both WT and transgenic Arabidopsis, including *AtPAL*, *AtCHS*, *AtCHI*, *AtF3H*, *AtF3*′*H*, *AtDFR*, *AtANS*, and *AtUF3GT*. The results showed that expression levels of all of the tested genes were significantly increased in two transgenic lines with *GmCSN5A* overexpression only under low P conditions, but not under high P conditions (Figure 5E).

Similarly, increases in fresh weight and anthocyanin content were also observed in *GmCSN5B* overexpression lines compared to those in WT only under Pi-deficient conditions, not under Pi-sufficient conditions (Figure 6B,C). However, P content in *GmCSN5B* overexpression lines was no different from that in WT at two P levels (Figure 6D). Furthermore, except *CHI*, expression levels of the tested genes were significantly increased in transgenic lines with *GmCSN5B* overexpression under low P conditions (Figure 6E). These results together suggest that both *GmCSN5A* and *GmCSN5B* play important roles in anthocyanin synthesis in plant responses to low P stress. Meanwhile, hypocotyl length was significantly decreased in Arabidopsis with overexpressing *GmCSN5B* after grown in the dark for 5 d (Figure S2). However, no difference in hypocotyl length was found between WT and plants with overexpressing *GmCSN5A* (Figure S2).

## 3. Discussion

Phosphorus is one of essential macronutrients and participates in numerous biochemical and metabolic processes [1,2]. Among the adaptive strategies of plants to P deficiency, increased anthocyanin accumulations in shoot/leaves are generally considered to improve plant tolerance to P deficiency, and widely observed in plants including Arabidopsis, tomato, tobacco, and maize [9,10,13,26]. In this study, enhanced anthocyanin accumulations in soybean young leaves and old leaves were also found under Pi-deficient conditions, as reflected by the fact that their values were 1.2- and 1.3-fold higher than those plants in Pi-sufficient conditions (Figure 1C). Furthermore, it was observed that anthocyanin content in old leaves was 12.7% higher than that in young leaves by Pi starvation (Figure 1C), suggesting that more anthocyanins were synthesized in old leaves by Pi starvation because Pi might be transported out of old leaves to active tissues/organs in plants. Consistently, P concentration in old leaves was 33.2% lower than that in young leaves at low P levels (Figure 1D). Similarly, higher anthocyanin content, accompanied with lower P concentration was also observed in shoot/leaves of other plants under low Pi stress, such as Arabidopsis and Medicago [7,44]. Accompanied with anthocyanin accumulations, expression levels of several genes related to anthocyanin synthesis were significantly up-regulated by Pi starvation in soybean old leaves, including *GmPAL*, *GmCHS*, *GmCHI*, *GmF3*′*H*, *GmDFR*, *GmANS*, and *GmUF3GT* (Figure 2). Meanwhile, it was widely observed that transcripts of one or more homologues related to anthocyanin synthesis were increased by P deficiency in plants, strongly suggesting that the regulatory pathway of Pi starvation responsive anthocyanin synthesis is well conserved in plants [7,12,23,26].

Consistently, we found that overexpressing *GmCSN5A* and *GmCSN5B* led to higher plant fresh weight, accompanied with more anthocyanin accumulations in plants under P-deficient conditions (Figure 5 and Figure 6), partially suggesting that increased anthocyanin synthesis could contribute to enhancing plant tolerance to P deficiency. It is generally believed that anthocyanin accumulations play a role in scavenging reactive oxygen species (ROS), and protecting chloroplasts from photoinhibitory damage in plants under abiotic stress conditions, such as N and P deficiencies [18,19,20,45,46]. For example, mutation of *ZmSRO1e* in maize enhanced anthocyanin accumulations and ROS scavenging activity, and thus led to higher plant biomass, suggesting that increased anthocyanin accumulations resulted in plant adaptation to Pi starvation through avoiding ROS inhibition [46]. However, there should be further analysis into whether anthocyanin accumulations in Arabidopsis with *GmCSN5A*/*B* overexpression could inhibit excess ROS accumulations under low P conditions, which is widely observed in plants subjected to Pi starvation [47].

Functions of *CSN5A* are generally considered to control plant photomorphogenesis in Arabidopsis, such as growth of hypocotyl and cotyledons [36,37,38]. For example, *csn5* mutants exhibited short hypocotyl and unexpanded cotyledons in seedlings grown in the dark [37,38]. However, hypocotyl length in Arabidopsis with overexpressing *GmCSN5B*, was significantly shorter than WT grown in the dark (Figure S2). These results strongly suggested that both increased and suppressed expression levels of *CSN5* could result in the abnormal growth of plant hypocotyls. However, overexpressing GmCSN5A does not have any effect on the hypocotyl length and root length. It might be caused by differences in amino acids between GmCSN5A and GmCSN5B, as reflected by a transmembrane domain presenting in GmCSN5B, not in GmCSN5A (Figure 3), which merit further studies.

In addition to influencing hypocotyl growth, increased anthocyanin accumulations were observed in loss-function mutants of *CSN5a*, strongly suggesting that CSN5A participates in mediating anthocyanin synthesis in Arabidopsis [36,37,38]. CSN5 was suggested to regulate anthocyanin accumulation via interactions with one of SCF complexes, SCF^COI1^ and its substrate, jasmonate ZIM-domain (JAZ) proteins, which negatively mediates the stability of a key transcription factor in anthocyanin synthesis, PAP1, and thus mediates expression levels of anthocyanin biosynthetic genes, including *DFR* and *UF3GT* [32,38,48]. Furthermore, it is generally considered that the anthocyanin synthesis is affected directly by transcription levels of several genes encoding enzymes in anthocyanin biosynthetic pathway in plants [7,23]. For example, *tt3*, a mutant of DFR which is the rate-limiting enzyme in anthocyanin biosynthesis, decreases anthocyanin content under normal growth conditions or low N stress [49]. In this study, overexpressing *GmCSN5A* and *GmCSN5B* in Arabidopsis led to anthocyanin accumulations in Arabidopsis only under P-deficient conditions, as reflected by values more than 1.4- and 1.2-fold higher than those in WT (Figure 5 and Figure 6), accompanied by increased transcripts of gene functions in anthocyanin synthesis, including *AtPAL*, *AtCHS*, *AtF3H*, *AtF3*′*H*, *AtDFR*, *AtANS*, and *AtUF3GT* (Figure 5 and Figure 6), strongly suggesting that *GmCSN5A*/*B*-mediated anthocyanin accumulations under Pi-deficient conditions. It has widely been observed that CSN5 exhibits non-identical functions by binding to different protein targets [35]. The functions of GmCSN5A/B mediating the anthocyanin synthesis in response to Pi starvation probably depend on their interacted proteins. Meanwhile, SPX4, a main regulator in P signaling network, has recently been suggested to transduce the Pi starvation signal to mediate anthocyanin synthesis in Arabidopsis through interacting with PAP1 [26]. It is speculated that *GmCSN5A/B* might be associated with SPX4-PAP1 complex to regulate anthocyanin accumulation in response to Pi starvation, which merits further analysis.

## 4. Materials and Methods

### 4.1. Plant Materials and Growth Conditions

Soybean genotype YC03-3 provided by Root Biology Center, South China Agricultural University, China was used in this study. The seeds were germinated for 4 d as described previously [50]. After germination, uniform seedlings were transplanted into nutrient solution containing 1500 μM KNO_3_, 400 μM NH_4_NO_3_, 1200 μM Ca(NO_3_)_2_, 25 μM MgCl_2_, 300 μM K_2_SO_4_, 500 μM MgSO_4_, 0.3 μM (NH_4_)_2_SO_4_, 0.5 μM CuSO_4_, 1.5 μM MnSO_4_, 1.5 μM ZnSO_4_, 0.16 μM (NH_4_)_6_Mo_7_O_24_, 40 μM Fe-Na-EDTA, 2.5 μM NaB_4_O_7_, and 250 μM (+P) or 5 μM (−P) KH_2_PO_4_. The pH value of nutrient solution was adjusted to 5.8, and the nutrient solution was replaced every 7 d. After 12 d of P treatments, as described previously [51], soybean fresh weight, content of P concentration and anthocyanin in the youngest trifoliate leaves (i.e., young leaves) and primary leaves (i.e., old leaves) were measured. All experiments included four replicates.

Arabidopsis Columbia (Col-0) ecotype and tobacco (*Nicotiana benthamiana*) were used as background of transformation in this study. The seeds of Arabidopsis and tobacco were obtained from Dr. Jinxiang Wang at South China Agricultural University, China. Arabidopsis and tobacco plants were grown in a greenhouse at 22–24 °C and 60% relative humidity with a 16 h/8 h light/dark photoperiod.

### 4.2. Analysis of P Concentration and Anthocyanin Content

To determine P concentration, about 0.1 g of plant dry samples were ground into powder and digested with H_2_SO_4_-H_2_O_2_. P concentration in the extracts was measured according to the method described by Murphy and Riley [52].

For anthocyanin content measurement, about 0.1 g of fresh leaf samples were ground into powder by liquid N_2_ and dissolved in extraction solution (0.1 M HCl mixed with 95% ethanol). The mixtures were incubated at 60 °C for 30 min. After centrifugation at 12,000 rpm for 15 min at 4 °C, the supernatants were collected and spectrophotometrically detected at 530 and 657 nm with a microplate spectrophotometer (Thermo Scientific, Waltham, MA, USA). Anthocyanin content was calculated as previously described [53].

### 4.3. Phylogenetic Analysis and Characterization of GmCSN5A/B in Plants

GmCSN5A (Glyma.04G075000) and GmCSN5B (Glyma.06G076000) were identified in soybean genome by BLAST searches using the AtCSN5A (At1G22920) and AtCSN5B (At1G71230) as the query sequences at the Phytozome website (http://www.phytozome.net, accessed on 10 February 2019). The phylogenetic tree was constructed using CSN5 proteins from soybean, Arabidopsis, common bean (*Phaseolus vulgaris*), Medicago, tomato, rice, and maize using MEGA 5.05 based on the Neighbor-Joining method [54]. Conserved domain analysis was performed at the SMART website (http://smart.embl.de/, accessed on 17 May 2020) as previously described [55].

### 4.4. Subcellular Localization Analysis of GmCSN5A/B

The subcellular localization of GmCSN5A and GmCSN5B was analyzed as previously described [43]. Briefly, the full-length of *GmCSN5A* and *GmCSN5B* was separately amplified and cloned into a *pEGAD* vector with eGFP at its C-terminus. The *GFP* and *GmCSN5A*/*5B*-*GFP* plasmids and the plasma membrane marker *AtPIP2A*-mCherry were co-transformed into tobacco leaves by *Agrobacterium*-mediated transformation. The fluorescence signals were observed in a Zeiss LSM7 Duo confocal microscope (Zeiss, Oberkochen, Germany) at 488 nm for GFP and 543 nm for mCherry.

### 4.5. Functional Characterization of GmCSN5A/B in Arabidopsis

The full-length of *GmCSN5A* and *GmCSN5B* was separately amplified and cloned into *pTF101s* vector according to the method described previously [43]. The *GmCSN5A*- and *GmCSN5B*-*pTF101s* plasmids were transformed into Arabidopsis by *Agrobacterium*-mediated transformation as described previously [50]. Two homozygous T3 lines (OX1/2/3/4) were separately selected and used for functional analysis. The transcripts of *GmCSN5A* and *GmCSN5B* in transgenic Arabidopsis were determined by quantitative RT-PCR (qRT-PCR). To investigate the functions of *GmCSN5A* and *GmCSN5B*, both wild type (WT, Columbia-0) and transgenic Arabidopsis seeds were cultivated on peat soils (Jiffy, Zwijndrecht, Netherlands) supplied with or without phosphorus. After 8 or 13 d growth, Arabidopsis plants were separately harvested for fresh weight, P and anthocyanin content analysis. All experiments had four biological replicates.

Furthermore, to investigate the functions of *GmCSN5A* and *GmCSN5B* in photomorphogenesis, both WT and transgenic Arabidopsis were germinated on solid Murashige and Skoog (MS) medium in the dark for 5 d. The length of hypocotyls and roots was analyzed with Image J (National Institutes of Health, Bethesda, MD, USA). All experiments had 19 biological replicates.

### 4.6. RNA Extraction and Quantitative RT-PCR Analysis

Total RNA was extracted from soybean and Arabidopsis plants using the RNAsolve reagent (OMEGA Bio-Tek, Norcross, GA, USA), and was then purified with RNase-free DNase I (Invitrogen, USA). About 1 μg of RNA was reversely transcribed to complementary DNA (cDNA) with MMLV-reverse transcriptase (Promega, Madison, WI, USA). qRT-PCR was performed using a SYBR Premix kit (Promega, Madison, WI, USA) on a Rotor-Gene 3000 real-time PCR system (Corbett Research, Mortlake, Australia). The *GmEF*-*1a* and A*tEF*-*1a* were separately used as an internal control. Relative expression of genes was calculated as the ratio of expression of the tested genes to those of *EF*-*1a* as described previously [56]. Specific gene primers are listed in Table S1.

### 4.7. Statistical Analysis

All data were analyzed using Microsoft Excel 2019 (Microsoft Company, Redmond, WA, USA) and SPSS program (v21.0; SPSS Institute, Chicago, IL, USA) for calculating mean, standard error, and Student’s *t*-tests.

## 5. Conclusions

In summary, two *CSN5* members in soybean were identified, and up-regulated by Pi starvation. Furthermore, overexpressing *GmCSN5A* and *GmCSN5B* in Arabidopsis resulted in significant increases in anthocyanin accumulation and fresh weight in shoot accompanied with increased transcripts of gene functions in anthocyanin synthesis including *AtPAL*, *AtCHS*, *AtF3H*, *AtF3*′*H*, *AtDFR*, *AtANS*, and *AtUF3GT* under P-deficient conditions. Taken together, these results suggest that Pi starvation responsive *GmCSN5A* and *GmCSN5B* may mediate anthocyanin accumulation in plants in responses to P deficiency.

## Data Availability

The sequences of *GmCSN5A* and *GmCSN5B* were acquired from http://www.phytozome.net, accessed on 10 February 2019. The data presented in this study are available in article and Supplementary Materials.

## Supplementary Materials

The following are available online at https://www.mdpi.com/article/10.3390/ijms222212348/s1.
