# Supplementary material for: Phosphate (Pi) Starvation Up-Regulated GmCSN5A/B Participates in Anthocyanin Synthesis in Soybean (Glycine max) Dependent on Pi Availability
Source: Int J Mol Sci. 2021 Nov 16;22(22):12348. doi: 10.3390/ijms222212348 (PMC8623310; doi:10.3390/ijms222212348)
Supplement: Supplementary file 1 [file ijms-22-12348-s001.zip › Supplementary files/Supplementary figures S1 to S2.pdf]

**Figure S1**

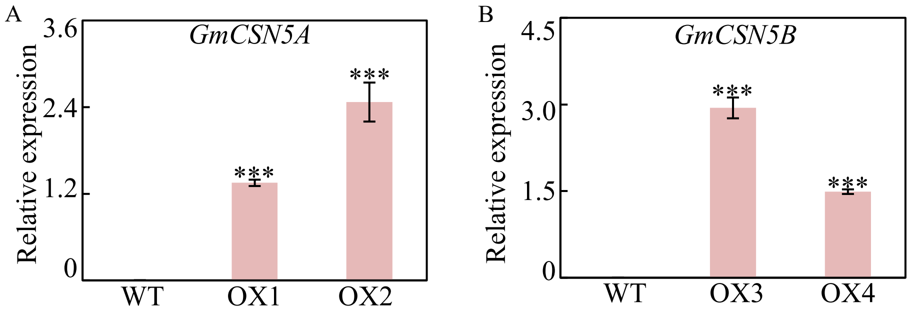


Figure S1. Verification of transgenic Arabidopsis plants with *GmCSN5A*/*B* overexpression. (A) Relative expression of *GmCSN5A* in overexpressing *GmCSN5A* Arabidopsis. (B) Relative expression of *GmCSN5B* in overexpressing *GmCSN5B* Arabidopsis. Asterisks indicate significant differences between WT and *GmCSN5A*/*B* transgenic Arabidopsis in Student's *t*-test: *** $P<0.001$.

**Figure S2**

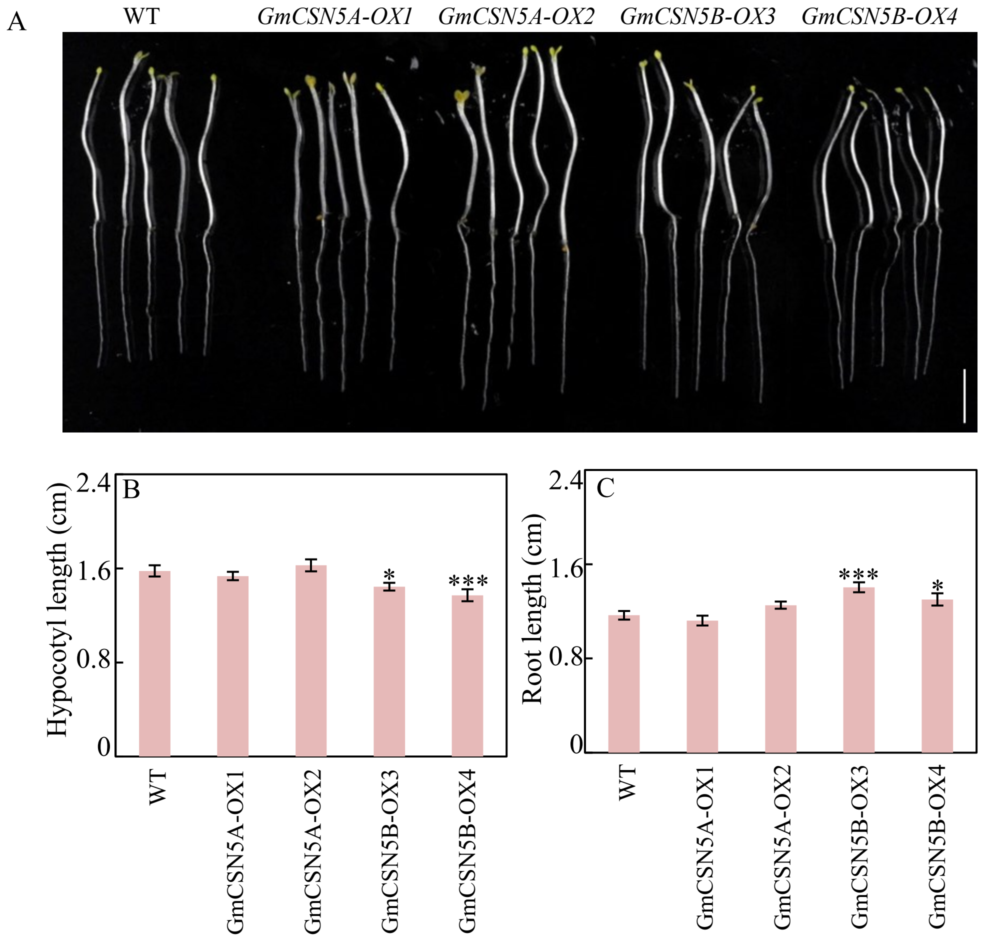


Figure S2. Effects of overexpressing *GmCSN5A*/*B* on Arabidopsis grown in the dark. (A) Phenotype of overexpressing *GmCSN5A*/*B* and WT Arabidopsis grown in the dark for 5 days. (B) Hypocotyl length. (C) Root length. Data are mean of 19 replicates ± SE. Asterisks indicate significant differences between WT and *GmCSN5A*/*B* transgenic Arabidopsis in Student's *t*-test: * $P$<0.05; *** $P$<0.001. Bars =0.5 cm.
